# Integrative Taxonomy and Synonymization of *Aculus mosoniensis* (Acari: Eriophyidae), a Potential Biological Control Agent for Tree of Heaven (*Ailanthus altissima*)

**DOI:** 10.3390/insects13050489

**Published:** 2022-05-23

**Authors:** Enrico de Lillo, Francesca Marini, Massimo Cristofaro, Domenico Valenzano, Radmila Petanović, Biljana Vidović, Tatjana Cvrković, Marie-Claude Bon

**Affiliations:** 1 Dipartimento di Scienze del Suolo, della Pianta e degli Alimenti, University of Bari Aldo Moro, Via Amendola, 165/a, 70126 Bari, Italy; domenico.valenzano@uniba.it; 2 Biotechnology and Biological Control Agency (BBCA) Onlus, Via Angelo Signorelli 105, 00123 Rome, Italy; fra.rini.bbca@gmail.com (F.M.); m.cristofaro55@gmail.com (M.C.); 3 Department of Entomology and Agricultural Zoology, Faculty of Agriculture, University of Belgrade, Nemanjina 6, 11080 Belgrade-Zemun, Serbia; rpetanov@agrif.bg.ac.rs (R.P.); magud@agrif.bg.ac.rs (B.V.); 4 Serbian Academy of Sciences and Arts, Knez Mihailova 35, 11000 Belgrade, Serbia; 5 Institute for Plant Protection and Environment, Department of Plant Pests, Laboratory for Molecular Diagnostics and Laboratory for Applied Entomology, Banatska 33, 11080 Belgrade-Zemun, Serbia; tanjacvrkovic@yahoo.com; 6 European Biological Control Laboratory, USDA-ARS, 810, Avenue du Campus Agropolis, 34980 Montferrier-sur-Lez, France; mcbon@ars-ebcl.org

**Keywords:** *Aculops taihangensis*, deutogyne, molecular analysis, *CO1*, ITS1, invasive plant species

## Abstract

**Simple Summary:**

Tree of heaven, *Ailanthus altissima*, is a deciduous tree indigenous to China and introduced for ornamental purposes into North America and Europe. It shows a highly invasive profile in field, urban, and suburban areas, posing a serious threat to ecosystems in the introduced ranges. The current management of this noxious infesting plant by mechanical and chemical measures remains ephemeral and incomplete. A multi-tactic approach emphasizing classical biological control appears highly relevant. Eriophyid mites are well known for their high specificity and for the impact caused to the associated hosts, resulting in relevant potential biological control agents of infesting plants. The partially unresolved taxonomy of the eriophyid mite species reported on the tree of heaven is currently seen as an impediment to their further concern as biocontrol agents. This paper investigated morphological and molecular characters of *Aculus mosoniensis* in order to better clarify its taxonomic status. A paratype specimen of *Aculops taihangensis* was also studied, and this mite species was reassigned to the genus *Aculus*. The strong congruence between morphological and molecular analyses for all mites collected on tree of heaven in Europe led to the conclusion that *A. mosoniensis* is a junior synonym of *Ac. taihangensis*.

**Abstract:**

The taxonomy of *Aculus mosoniensis* appears to be an unresolved question and its clarification is required, owing to the potential relevance of this mite species as a biological control agent of the tree of heaven. This paper is aimed at giving accurate details on a previously and shortly announced synonymization with *Aculops taihangensis,* using a morphological and molecular approach. A fusiform morph of *A. mosoniensis* was distinguished from a vermiform morph and this latter was recognized as deutogyne, which was herein documented. Phylogenetic relationships between Chinese *Ac. taihangensis* and all *A. mosoniensis* mites collected in twenty localities in Europe were examined through the analysis of the mitochondrial cytochrome c subunit I (*CO1*) protein and the nuclear ribosomal internal transcribed spacer 1 region (ITS1). *CO1* sequences of *Ac. taihangensis* from the Shandong province in China and those from mites collected in Austria and Slovenia were 100% identical; the ITS1 sequence of an *Ac. taihangensis* paratype matched for 99.8% with those obtained from protogynes and deutogynes of *A. mosoniensis* collected in Italy. All these data supported the announced synonymization of *A. mosoniensis* with *Ac. taihangensis*. *Aculus* *mosoniensis* was found genetically variable, with five *CO1* haplotypes in Europe (becoming eight along with those of *Ac. taihangensis*) clustering in two highly supported maternal lineages and eight ITS1 haplotypes (becoming nine along with those of *Ac. taihangensis*) distributed in four supported clades. No overlap between intra- and interspecies distances was observed for both markers and all studied *A. mosoniensis* populations clustered in one monophyletic mitochondrial clade, suggesting that only one single species might occur in Europe. However, more mite clades may be related to more tree of heaven biotypes with potential ecological differences, which might have potential effects on the biological control and should be investigated.

## 1. Introduction

The tree of heaven (ToH), *Ailanthus altissima* (Mill.) Swingle, belongs to Simaroubaceae (Sapindales), which is a family of Eastern Asian origin [1]. *Ailanthus altissima* is a deciduous tree species, alien in Europe and North America, and considered as one of the most invasive and noxious infesting plants worldwide favored by its bioecological characteristics [2,3,4,5]. Eriophyid mites (Acari: Eriophyoidea) are highly specialized plant feeders, and most of them are well known for their high specificity and for the impact they have on the associated hosts, despite their extremely small size [6]. *Aculops ailanthi* Lin, Jin & Kuang, *Aculops taihangensis* Hong & Xue (the new combination of the genus name was assigned only at the end of this paper), *Aculus altissimae* Xue & Hong, and *Aculus mosoniensis* (Ripka) have been found associated with *A. altissima* [7,8]. All of them have been recorded in China, except for *A. mosoniensis*, which has been reported only in European countries [6], and *Ac. Ailanthi,* also found in the USA [9,10]. *Aculus mosoniensis* and *Ac. ailanthi* are considered very promising biological control agents based on the thus-far records of impact noticed to provoke on their host [6,9,10]. Host-range testing under open-field conditions recently carried out in a European context provided a good indication of the specificity of *A. mosoniensis* for ToH [6]. As reported in an earlier paper [7], the taxonomic and the bioecological characterization of *A. mosoniensis* has been under evaluation, and the synonymy between *Ac. taihangensis* and *A. mosoniensis* was announced in a short review paper with a very brief and non-exhaustive explanation.

Different procedures of specimen slide mounting can stress the morphology and hide some fundamental details, especially in such tiny eriophyid mites, which can lead to poor descriptions of species and need for their revision [11]. Integrative taxonomy, as coined by Dayrat [12] by combining molecular phylogenetic and phylogeographical information, was demonstrated to be a valid approach to support the taxonomy of eriophyid mites [13,14,15]. A further interference in the correct definition of the taxonomic status of an eriophyid species can come from its life strategy. Overwintering deutogyne morphs, which are characterized by an innate resistance to the environmental stresses, can have a distinctive morphology in respect of spring–summer protogyne morphs [16]. The differences can be noticeable but very pale for some species and require careful morphometric examination [17]. Therefore, in the definition of the taxonomic status of an eriophyid mite, it is worthy of interest to examine the biological cycle in order to verify the existence of these morphs, which can make the identification less easy and lead to the constitution of species which might need, then, to be reassigned and synonymized. This is the case of the eriophyoid species currently known to be associated with ToH: some species have a poor description and no biological data are available. This means that synonymy of *A. mosoniensis* with other species might be possible.

The taxonomic status of *A. mosoniensis,* as well as its ecological relationship with the host plant, appear to be unresolved questions and their clarification is required considering the potential relevance of this mite species as a biological control agent for ToH. Therefore, this paper is aimed at elucidating the taxonomic position of *A. mosoniensis* using morphology and molecular characters, verifying and explaining accurately the announced synonymization along with *Ac. taihangensis*.

## 2. Materials and Methods

### 2.1. Morphological Study by Light Microscopy

Occasional samplings of ToH were carried out from 2016 to 2017 in Apulia (Bari, Italy, 41°06′38″ N, 16°52′55″ E; 19 m a.s.l.) and Lazio (Saxa Rubra, Rome, Italy, 41°58′52″ N, 12°29′38″ E; 18 m a.s.l.). Samples were transported to the laboratory and eriophyid mites were examined under a light microscope. Specimens of *A. mosoniensis* were slide-mounted according to the protocol described in Baker et al. [18] and Keifer [19]. Only one paratype specimen of *Ac. taihangensis* preserved in absolute ethanol (tube n. 24, 22 August 2003, Taihang mountain, Hebei province, China, legit Xue Xiao-Feng) was mounted on a slide following the procedure described by Keifer [19].

The terminology and setal notation used in the morphological description of the mites follow mainly those proposed by Lindquist [20]. All morphological measurements were taken using a phase contrast microscope Olympus BX50 according to Amrine and Manson [21], as modified by de Lillo et al. [11], and are given in micrometers (μm). Measurements are rounded off to the nearest integer, referring to the length of the morphological traits unless specified otherwise, and express the range of values of the studied population. Line drawings were handmade through a camera lucida according to de Lillo et al. [11], and figure abbreviations mainly follow Amrine et al. [22].

### 2.2. Morphological Study by Scanning Electron Microscopy

For scanning electron microscopy observations, leaf and bud samples of ToH were collected in Belgrade, Serbia (44°50′33″ N, 20°24′19″ E, 82 m a.s.l.), on 2 May 2017 (for *A. mosoniensis* protogynes) and 27 February 2018 (for *A. mosoniensis* deutogynes), respectively; further bud samples were collected in Bari, Italy (41°06′38″ N, 16°52′55″ E; 19 m a.s.l.), on 16 December 2019 (for *A. mosoniensis* deutogynes). Live mites were collected with a fine entomological needle from fresh plant parts under a stereomicroscope and transferred on aluminum holders. For Serbian specimens, prior to the observation, holder-mounted mites were gold-coated (BAL-TEC SC-RD005) by a sputter coater (BAL-TEC AG, Balzers, Liechtenstein) for 100 s under 30 mA ion current. Micrographs were recorded by the scanning electron microscope JSM-6390 LV (JEOL GmbH, Munich, Germany) at the Laboratory of Electron Microscopy, Faculty of Agriculture, University of Belgrade (UBS), Serbia. For Italian specimens, holder-mounted mites were treated under a critical point dryer (Leica EM CPF300, Wetzlar, Germany) and gold-coated by a sputter coater (Edwards S150A, Irvine, CA, USA) for 60 s under 30 mA ion current. Micrographs were recorded by scanning electron microscopy (TM3000; Tabletop Microscope, Hitachi, Chiyoda, Japan) at SELGE Laboratory, Dipartimento di Scienze del Suolo, della Pianta e degli Alimenti, University of Bari Aldo Moro (UBI), Italy.

### 2.3. Sampling for the Molecular Study and DNA Extraction

Live mites were taken directly from ToH leaves in twenty localities distributed across ten European countries, as well as one paratype specimen of *Ac. taihangensis* (Table 1). All mite specimens were preserved in 96% ethanol in collecting tubes and stored at 4 °C until DNA analysis. The DNA extraction, PCR amplification, and sequencing of all specimens were performed by three different laboratories, i.e., Department of Plant Pests, Institute for Plant Protection and Environment (IPPE, Belgrade, Serbia), the USDA-ARS European Biological Control Laboratory (EBCL, Montpellier, France), and UBI.

At IPPE, genomic DNA was extracted from a pool of 5–10 specimens using DNeasy Blood & Tissue Kit (Qiagen GmbH, Hilden, Germany), according to the manufacturer’s instructions, with modifications based on Dabert et al. [23].

At EBCL, genomic DNA was extracted from a pool of 5–7 specimens using a different method, as described by Kashefi et al. [24].

At UBI, total genomic DNA was extracted from single specimens by adding 100 µL of Chelex suspension (10% in water) (InstaGene matrixTM—BioRad, Hercules, CA, USA) and 5 µL of proteinase-K (20 mg/μL) (Qiagen). More specifically, the UBI extracted DNA from live mites collected directly from leaves (six protogynes and/or males with fusiform aspect) and from overwintering buds (nine deutogynes with vermiform aspect), as well as from a second paratype specimen of *Ac. taihangensis*. These mites were individualized in tubes, spun at 10,000 rpm for 10 min at 4 °C, and stored at −20 °C until DNA extraction.

All residual DNA are archived at the place of the DNA extraction, except at UBI where there was no DNA left post-amplification of ITS1, hence preventing the amplification of the barcode region.

### 2.4. Markers for the Molecular Study

The barcode region of the cytochrome oxidase subunit I gene (*CO1*) was amplified at IPPE and EBCL as described in Marini et al. [6] and in Kashefi et al. [24], respectively. The amplified fragments were sequenced in both directions with the same primer pairs as in the initial PCR procedure, using ABI technology by Macrogen (Seoul, Korea) for IPPE and Genoscreen (Lille, France) for EBCL. Genbank accession numbers of *COI* sequences are given in Table 1.

The internal transcribed spacer 1 region of ribosomal DNA (ITS1) primarily targeted by UBI was obtained using the primer pair 18S/5.8S reported in Navajas et al. [25]. PCRs were performed in 20 μL final volume containing 10 μL of Go Taq^®^ Green Master Mix 2X (Promega), 1 μL for each primer 10 μM, 3 μL of water, and 5 μL of DNA template. Touch-down PCR reactions were conducted following Carew et al. [26]. Five μL of amplicons were purified using Illustra™ ExoProStar™ (GE Healthcare Life Sciences) according to the manufacturer’s instructions and sequenced in both senses using ABI technology by Applied Biosystems™ DNA Analyzer 3730XL (Genomic Facility at Foundation Edmund Mach San Michele all’Adige, Trento, Italy).

At IPPE and EBCL, we followed the same procedure as described above except that (i) the DNA template was always diluted, (ii) the PCR composition, apart from the primers, was similar to that used for the barcode, and (iii) sequencing was performed by different companies. The ITS1 products obtained in Colombes3 (France) and TOHA2 (Italy) were further purified using Qiaquick gel extraction kit (Qiagen, Valencia, CA) and sequenced after cloning into the pGEM-T easy vector (Promega, Madison, WI, USA). Sequencing of 10 clones for each ITS1 product was carried out using the universal M13 primers. Genbank accession numbers of ITS1 sequences are given in Table 1.

### 2.5. Alignments, Phylogenetic Analyses, and Genetic Distance for the Molecular Study

Both strands of each amplicon were assembled into contigs using either FinchTV v.1.4.0 (www.geospiza.com (accessed on 1 April 2022) at IPPE or Bioedit version 7.2 [27] at EBCL and UBI. The *CO1* sequences were translated into amino acids to check for the absence of stop codons and frame shifts in this protein coding gene. Direct sequencing of ITS1 resulted in few sites that were heterozygous for single nucleotide polymorphisms in four specimens. The haplotypic phase of these heterozygous genotypes was inferred using PHASE as implemented in DnaSP v6 [28]. With respect to ITS1 clones, only clones represented at least twice were selected, to avoid inclusion of spurious mutation raised from PCR and cloning error which can produce a false spectrum of genomic diversity. Our dataset of 24 *CO1* sequences was then merged with twelve sequences of *Ac. taihangensis* retrieved from the Barcode of Life system (BOLD) and reported by Yin et al. [29]. A total of 36 *CO1* and 45 length-variable ITS1 sequences (combining clones and phased genotypes) were aligned by CLUSTAL W, as implemented in MEGA 11 [30]. We obtained a final *CO1* ingroup alignment of 658 characters, 79 of which were parsimony informative, and a final ITS1 ingroup alignment of 440 characters (after excluding gaps), 29 of which were parsimony informative. Identical sequences of *CO1* and ITS1 were merged to unique haplotypes with DnaSP v6. For both markers, we calculated pairwise genetic distances within *A. mosoniensis* and between *A. mosoniensis*, *Ac. Taihangensis,* and congeners using MEGA 11. We calculated both the K2P distance [31] (since it has often been used in the literature) and the uncorrected *p*-distance (since K2P could be inappropriate when employed for closely related taxa) [32]. Phylogenetic relationships were inferred in a maximum likelihood framework and 1000 bootstrap replicates in MEGA 11, and in a Bayesian framework in MrBayes 3.2 [33], with two independent analyses of four Markov chains calculated for 1 million generations, and a 25% “burn-in”. The most appropriate model of evolution selected based on the Bayesian information criterion (BIC [34]), as implemented in MEGA 11, was HKY + G [35] and JC [36] for *CO1* and ITS1, respectively. As *a priori* outgroups for the phylogenetic analyses were chosen: *Aculus ichnocarpae* (Ghosh & Chakrabarti) (Genbank accession number KM111094.1) and *Aculus amygdali* Xue & Hong (KM111095.1) for *CO1*, and *Aculus cercidis* (Hall) (KJ209712.1) for ITS1.

## 3. Results

### 3.1. Morphological Study

Winter and spring–summer populations of *A. mosoniensis* were observed and studied. The descriptions of males and spring–summer females were previously given [7,8]. A mixed population of fusiform and vermiform mites was observed on leaves collected during autumn (Saxa Rubra, 13 November 2015) in a preliminary collection. The first morph was composed of few females and males, whereas the second one was composed only of females. A further check was made on the mite population recovered into the buds (Saxa Rubra, 21 February 2017) and only vermiform females were found in them. During spring and summer, vermiform females were not found anymore. Therefore, the vermiform was retained to be the deutogynes of *A. mosoniensis*. Deutogynes were also collected in Bari (Italy) and Serbia. The morphology of the Italian deutogyne is reported below.

### 3.2. Description

**Figure 1:**
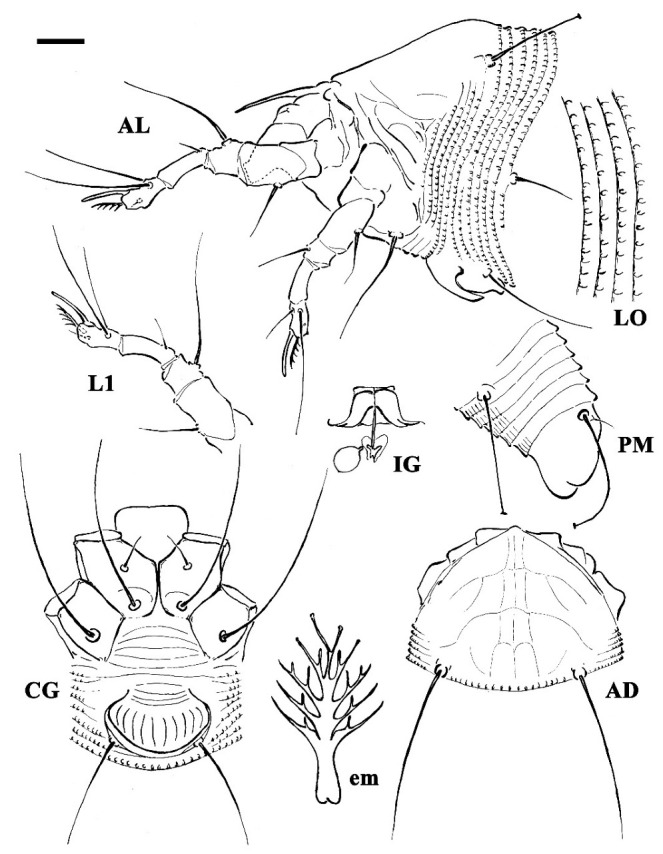
Line drawings of a deutogyne of *Aculus mosoniensis*. AD: prodorsal shield; AL: lateral view of anterior body region; CG: female coxigenital region; em: empodium; IG: internal female genitalia; LO: lateral view of annuli; L1: leg I; PM: lateral view of posterior opisthosoma. Scale bar: 10 μm for AD, AL, CG, IG, and PM; 5 μm for LO and L1; 2.5 μm for em.

**Figure 2:**
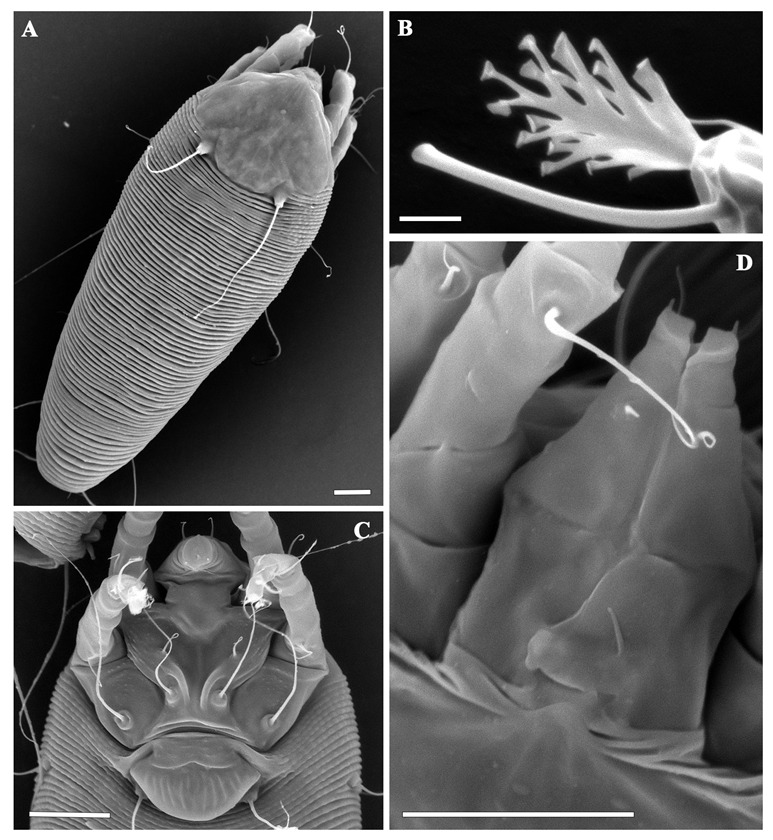
Scanning electron micrographs of deutogynes of *Aculus mosoniensis*: (**A**) dorsal view; (**B**) leg tarsal empodium and solenidion; (**C**) coxigenital region; (**D**) dorsal view of gnathosoma and frontal lobe. Scale bars: 10 μm for (**A**,**B**,**D**); 2 μm for (**B**).

**Figure 3:**
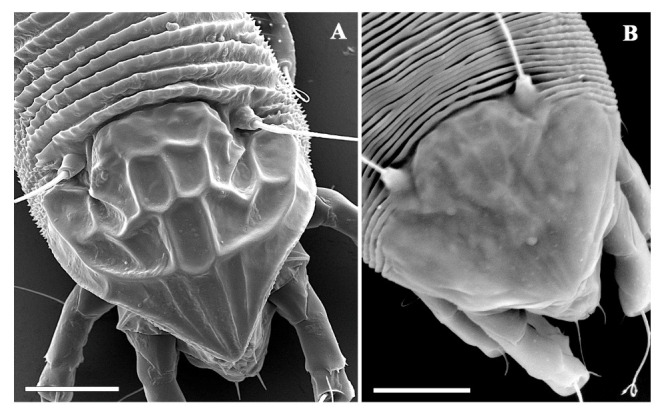
Scanning electron micrographs of a protogyne (**A**) and a deutogyne (**B**) of *Aculus mosoniensis*. Prodorsal shield and part of dorsal opisthosoma. Scale bars: 10 μm.

**DEUTOGYNE**: (n = 18 measured specimens) (Figure 1, Figure 2 and Figure 3). Body vermiform, 194–292 (including gnathosoma), 42–56 wide, 42–60 thick. **Gnathosoma** 21–25 projecting downwards, pedipalp coxal setae *ep* 2–3, dorsal pedipalp genual setae *d* 2–3, unbranched, pedipalp tarsus setae *v* undetectable, cheliceral stylets 16–19. **Prodorsal shield** 35–40, including the frontal lobe, 35–45 wide; frontal lobe 2 over gnathosomal base, anteriorly rounded in dorsal view. Network shield pattern often obscure and pale, composed of lines weaker and thinner than that of the protogyne: complete median line and complete pair of admedian lines connected by three transversal lines forming four pairs of cells; two pairs of transverse lines extending outermost from the admedian lines on the half and on the anterior 1/3 of the shield. Tubercles *sc* quite rounded, on the rear shield margin, 22–26 apart, scapular setae *sc* 36–43, directed backward. **Leg I** 41–46, femur 13–15, genu 6–7, tibia 13–13, tarsus 9–10, solenidion *ω* 9–10, quite straight, distally rounded, empodium 6–7, simple, 5-rayed; femoral setae *bv* 11–14, genual setae *l′′* 23–28, tibial setae *l′* 4–7, tarsal setae *ft′* 21–23, setae *ft′′* 24–29. **Leg II** 36–42, femur 12–14, genu 6–7, tibia 10–11, tarsus 9–10, solenidion *ω* 9–10, quite straight, distally rounded, empodium 6–7, simple 5-rayed; femoral setae *bv* 11–13, genual setae *l′′* 6–7, tarsal setae *ft′* 5–8, setae *ft′′* 24–32. **Coxae** I and II smooth; setae *1b* 10–14, tubercles *1b* 9–11 apart, setae *1a* 32–45, tubercles *1a* 6–7 apart, setae *2a* 51–63, tubercles *2a* 20–24 apart, prosternal apodeme 9–11. **Opisthosoma** dorsally arched with 66–76 dorsal semiannuli mainly smooth or with very weak and not-well-delimited microtubercles; 67–80 ventral semiannuli, with rounded microtubercles close to rear margin; 5–6 smooth semiannuli between coxae and genital coverflap; last 5 ventral and dorsal semiannuli with elongated microtubercles. Setae *c2* 17–26, on ventral semiannulus 8–10; setae *d* 52–83, on ventral semiannulus 23–27; setae *e* 22–27, on ventral semiannulus 42–48; setae *f* 36–47, on ventral semiannulus 63–75, 5 annuli after setae *f*. Setae *h2* 62–150 (the variability depends on the age of the mites—the oldest are those on the leaves in early spring and showed longer *h2* setae in respect to those collected from the buds in the previous winter), setae *h1* 4–5. **Genital coverflap** 10–14, 21–25 wide, coverflap with 8–10 longitudinal striae, setae *3a* 20–28, 14–17 apart; 3 close lines at genital coverflap base.

Basic morphological differences between protogyne and deutogyne are reflecting in more pronounced ornamentation of prodorsal shield in protogyne and in appearance of dorsal opisthosoma that is supplied with elliptical elongate microtubercles in protogynes and is mainly smooth or with very weak and not-well-delimited microtubercles in deutogynes. This is in accordance with the description of most of the deutogynes, and the explanation is in connection with the reduction of the body surface due to the desiccation during unfavorable winter conditions. Number of empodial rays and the genital striae are the same in both female morphs (Figure 2 and Figure 3).

**Figure 4:**
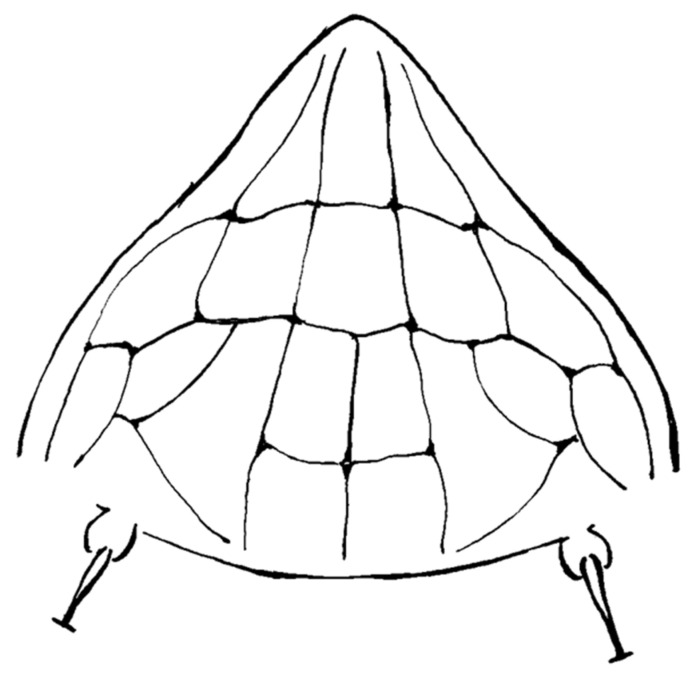
Line drawing of the prodorsal shield of *Aculops taihangensis* paratype.

A detailed morphological study of *Ac. taihangensis* was not possible owing to the few specimens comprising the ethanol’s preserved paratypes, as well as the strong tissue fixation induced by ethanol, which were unsuitable for a tissue digestion. However, it was possible to check the morphology of the prodorsal shield of a single female paratype, and the anterior lobe resulted to be rounded (Figure 4). The pattern of the prodorsal shield is characterized by a short median line on the rear half of the shield, a complete pair of admedian lines, two transverse lines joining the median with the admedian lines on the rear half of the shield forming four cells in the middle field of the shield, and a transverse line joining the admedian lines on the anterior one third of the shield forming two median cells; this transverse line continues in a pair of outer and arched submedian lines contributing to the delimitation of further cells between the submedian lines and the anterior-lateral edge of the shield (Figure 4). The original description of *Ac. taihangensis* indicates the presence of a complete median line [37], but a weak or obscure median line has also been observed in the anterior half of the shield in some specimens of the currently studied population of *A. mosoniensis*.

### 3.3. Molecular and Phylogenetic Analyses

**Figure 5:**
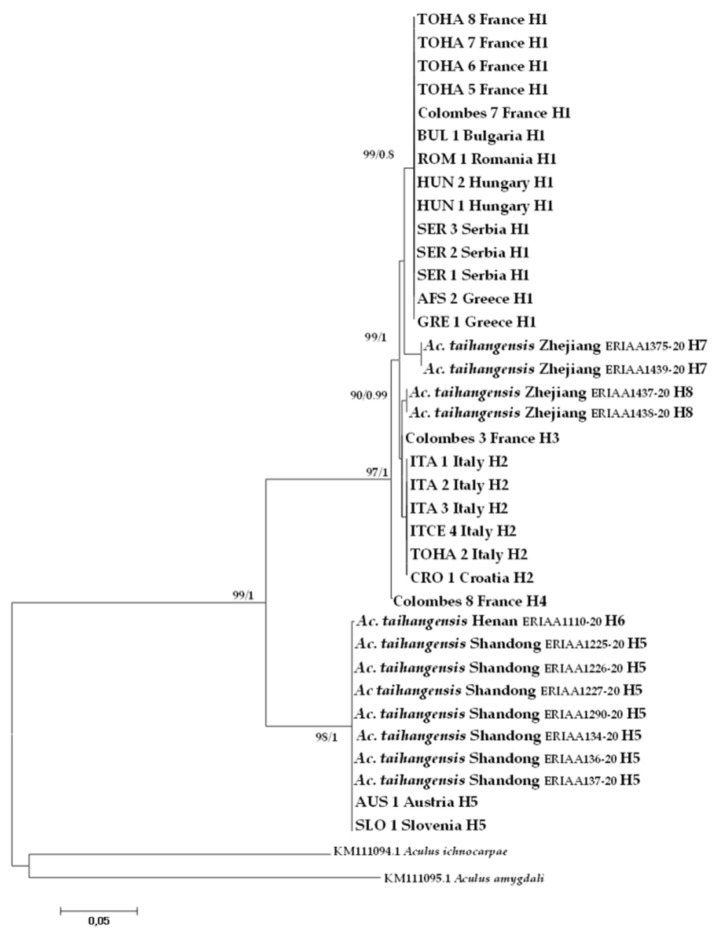
Maximum likelihood phylogenetic tree of *CO1* sequences of *Aculus mosoniensis*, *Aculops taihangensis,* and two congeners, *Aculus ichnocarpae* and *Aculus amygdali,* used as outgroups. Only significant values of bootstrap support (>90%) and of Bayesian posterior probabilities obtained by MrBayes (>0.8) are shown at the nodes. Branch lengths represent expected substitutions per site. The scale bar indicates the expected number of substitutions per site. Tip labels refer to specimen IDs and haplotypes listed in Table 1.

Barcode-compliant sequences of 658 nt in length were generated from all studied specimens (Table 1). A total of eight haplotypes were recovered from the *CO1* dataset combining *A. mosoniensis* and *Ac. taihangensis* sequences and the most common haplotype H1 was shared between six European countries (Figure 5). The haplotype H5 was harbored both by *Ac. taihangensis* from the Shandong province and *A. mosoniensis* mites from Austria and Slovenia. The pairwise genetic *p*-distance between *A. mosoniensis* and *Ac. taihangensis* averaged 7.68%, which is less than half the pairwise distances (23.03% and 24.78%) estimated between *A. mosoniensis*, *A. ichnocarpae,* and *A. amygdali*, respectively (Table 2). The phylogenetic reconstruction obtained under maximum likelihood and in a Bayesian framework (tree not shown) with *A. ichnocarpae* and *A. amygdali* as outgroups (Figure 5) demonstrated that all *A. mosoniensis* grouped with *Ac. taihangensis* in one fully supported clade with 99% support. It also demonstrated that this clade split into two major maternal lineages with high bootstrap support (97 to 98%), one including *Ac. taihangensis* from Shandong and Henan provinces and *A. mosoniensis* from Austria and Slovenia, the other clustering all the other European *A. mosoniensis* with *Ac. taihangensis* from the Zhejiang Province.

**Figure 6:**
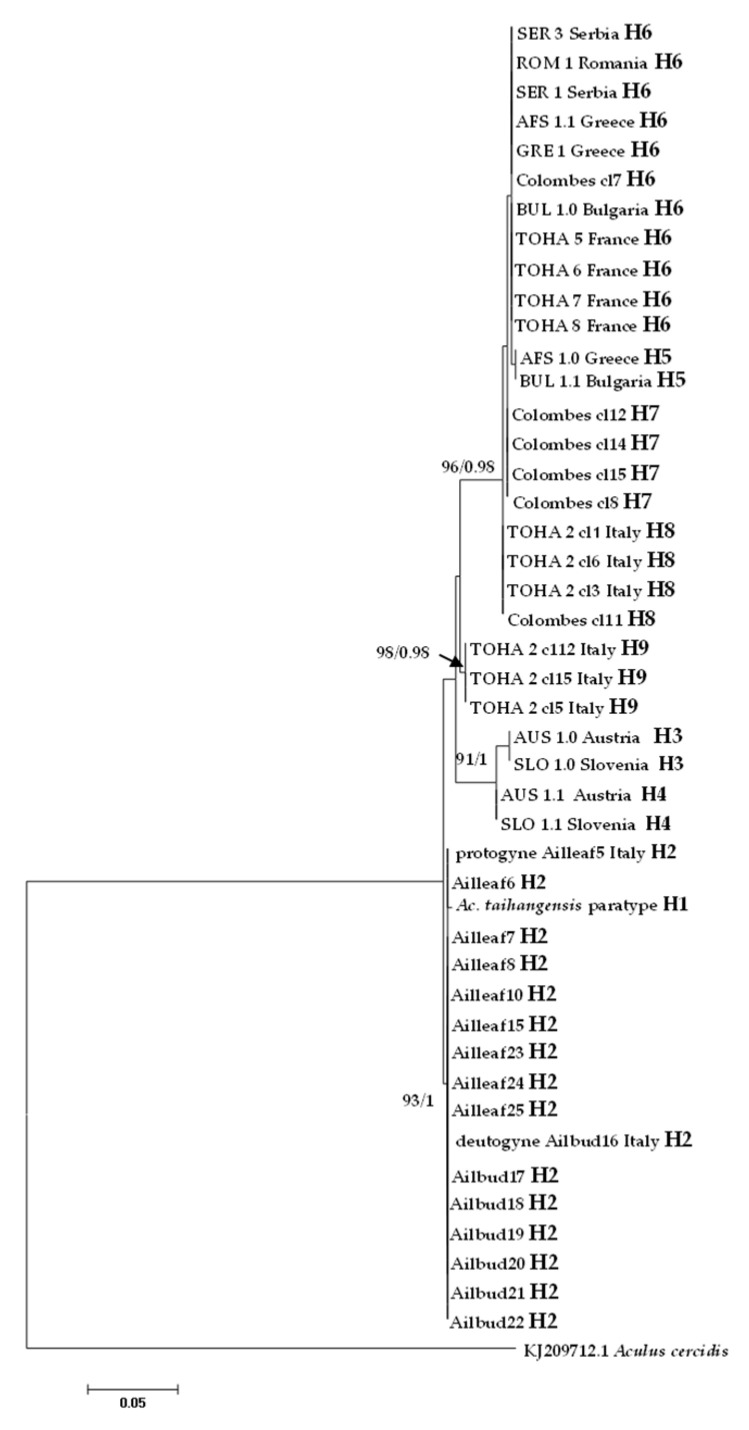
Maximum likelihood phylogenetic tree of *Aculus mosoniensis* ITS1 sequences, *Aculops taihangensis,* and one congener, *Aculus cercidis,* used as outgroup. Only significant values of bootstrap support (>90%) and of Bayesian posterior probabilities obtained by MrBayes (>0.9) are shown at the nodes. The scale bar indicates the expected number of substitutions per site. Tips labels refer to specimen IDs and haplotypes listed in Table 1.

In the ITS1 alignment generated from all analyzed specimens, due to the presence of indels, sequences varied in length from 440 bp (Colombes in France) to 449 bp (Austria and Slovenia), while ITS1 was 447 bp long in *Ac. taihangensis* paratype (Table 1). A total of nine haplotypes were recovered and the most common haplotype H6 was shared between five European countries (Figure 6).

All mites collected from leaves and buds at Bari (Italy) harbored the same ITS1 haplotype H2 which was shown to be the most closely related to H1 found in the Chinese *Ac. taihangensis* specimens, with a pairwise *p*-distance of 0.24%. The pairwise *p*-distances between all *A. mosoniensis* and *Ac. taihangensis* averaged 2.26%, which is almost sixteen times less than the pairwise distance (37.10%) estimated between *A. mosoniensis* and *A. cercidis* (Table 3). The phylogenetic reconstruction obtained in a maximum likelihood framework as well as in a Bayesian framework (tree not shown) with *A. cercidis* as an outgroup (Figure 6) demonstrated that most leaf nodes of the tree were highly supported, contrary to internal nodes. All *A. mosoniensis* collected in Bari (Italy) grouped with *Ac. taihangensis* in one fully supported clade with 93% support. Three other clades with high bootstrap support (91 to 98%) were evidenced: one gathering H3 and H4 from Austria and Slovenia, one with haplotype H9 found in Rome (Italy), and the last ones clustering four haplotypes (H5, H6, H7, and H8) widely distributed in Europe.

## 4. Discussion and Conclusions

The examination of the anterior prodorsal shield lobe of an *Ac. taihangensis* paratype allowed assigning this species to the genus *Aculus*, as previously suspected based on the original description [7]. A different procedure applied in digesting and mounting mites allowed us to revise the description of its prodorsal shield pattern which appears to be consistent with that of *A. mosoniensis*. All the other traits were already stated to be very similar between the two species [7]. The description of a deutogyne morph, as found in the current study, has also been added to the original description.

The two molecular markers applied in the present study resulted as congruent with the morphological examination and supported the similarity between the two species names. The *CO1* sequences of *Ac. taihangensis* from the Shandong province in China were identical to that of *A. mosoniensis* specimens collected in Austria and Slovenia, and the pairwise genetic *p*-distance between *Ac. taihangensis* and *A. mosoniensis* averaged 7.68%, which is much higher than the average intraspecific divergence (1.15%) in the Eriophyidae [29] but twofold less than estimates of intraspecific variation in other eriophyid mites such as *Aceria tosichella* Keifer (14.5%, e.g., [15]). Moreover, this estimate was also less than half the genetic distances between *Aculus* species (23–24.78%) estimated in the present study, in the Eriophyidae (19.86% [29]), and between congeneric species in other genera, such as *Aculodes* (20.2–21.5% [13]) or *Aceria* (15.6–22.0% [15]). In addition, the ITS1 sequence of *Ac. taihangensis* was found nearly identical (99.8%) to the ITS1 of *A. mosoniensis* specimens collected in Bari, Italy, and the pairwise genetic *p*-distance between *Ac. taihangensis* and *A. mosoniensis* averaged 2.6%, which was one order of magnitude less than the divergence between *A. mosoniensis* and the outgroup *A. cercidis* (37.10%). This latter estimate was surprisingly higher than the interspecies divergence reported for nuclear markers in other eriophyids, which ranged from 2% in *Aceria* [15] to 17% in *Abacarus* [38], but rather comparable to between *Abacarus* and *Aceria* [38]. A more accurate estimation of the range of divergence between *Aculus* species would require further investigation by increasing the ITS1 dataset of this genus as there is only one NCBI publicly available ITS1 sequence. Interestingly, in our analysis, *A. mosoniensis* was shown to exhibit some genetic polymorphism by uncovering five *CO1* in Europe (becoming eight along with those of *Ac. taihangensis*) and clustered in two major maternal lineages that were consistent but not entirely identical to the four clades/subclades described by the ITS1. The maternal lineage (Austria and Slovenia) and the one grouping most of the European populations were also evidenced in the ITS1 phylogenetic reconstruction. The large distribution of the latter clade suggested that most European countries have been colonized by mostly one *CO1* haplotype (H1) and one ITS1 haplotype (H6). Results also evidenced that the two European *A. mosoniensis* lineages were closely related to different *Ac. taihangensis* haplotypes found in two geographically distant provinces in China, i.e., Zhejiang and Shandon–Henan, suggesting two different origins in China of the European *A. mosoniensis*.

Based on the new morphological and molecular data, *A. mosoniensis* has to be considered a junior synonym of *Aculus taihangensis* new combination, which also means that the distribution range of *A. taihangensis* is not only restricted to China but also includes Europe.

Nevertheless, the eriophyid fauna associated with ToH still require further investigations. In fact, the prodorsal shield of *A. taihangensis* new. comb. appears to match that of *Ac. ailanthi*, as recently documented by Skrvarla et al. [10]. Unfortunately, the original morphological description of the latter species [39] is not consistent with the usual standard and shows some differences with *A. taihangensis* new. comb. [11]. Hence, it requires a better morphological characterization of the types in order to provide more careful details of the prodorsal shield pattern, genital, and coxal region, and morphometric data as well. Similarly, no molecular marker of this species has been currently known. Clarifying the taxonomic status of these two species is particularly important in the perspective of a biological control approach against ToH, especially because recent studies have shown a relevant impact of *Ac. ailanthi* on the ToH, as well as some similarities with *A. taihangensis* new. comb. concerning the host plant relationships [10,12]. In addition, deep investigations are needed to verify the real impact of the different haplotypes on the ToH.

Partial descriptions of new species, often out of standard, are confusing. The proper taxonomic identification of the natural enemies associated with a weed is a binding step of a weed biological control program. Due to some of their attributes, such as high host specificity and potentially high impact on their host, the general interest in the use of eriophyid mites in biological control of weeds is increasing [6]. It is precisely in this perspective that the current study represents an important step forward in the process of clarifying the eriophyid fauna associated with a highly invasive species such as ToH.

## Figures and Tables

**Table 1:** Collection information of the *Aculus mosoniensis* populations and *Aculops taihangensis* specimens analyzed, and Genbank accession numbers (id = unique numbers for DNA extraction vouchers).

Country	Locality/Province for China	GPS Coordinates	Collection Date (dd/mm/yyyy)	ID	CO1 GenBank Accession	CO1 Haplotype	ID	ITS1 GenBank Accession	ITS1 Haplotype
***Aculops taihangensis***									
China	Shandong	36°15′18″ N 117°00′36″ E and 37°31′37″ N 121°22′01″ E	21/07/2020		ERIAA1225–1227; 1290; 134; 136–137	H5			
					(Yin et al., 2022)				
	Henan	34°27′46″ N 113°04′26″ E	15/07/2020		ERIAA1110	H6			
					(Yin et al., 2022)				
	Zhejiang	29°16′37″ N 121°03′14″ E	30/08/2020 and 20/08/2020		ERIAA1375; 1439	H7			
		and 30°06′39″ N 118°54′03″ E			(Yin et al., 2022)				
	Zhejiang	30°06′39″ N 118°54′03″ E	20/08/2020		ERIAA1437–1438	H8			
					(Yin et al., 2022)				
	Taihang mountain, Hebei province	*na*	22/08/2003				Tube n. 24, Paratype, legit Xue Xiao-Feng	OM948701	H1
								(this study)	
***Aculus mosoniensis***									
Italy	Saxa Rubra, Rome	41°58′52″ N 12°29′38″ E	13/11/2015	ITA 1 ^a^	OM912723	H2			
					(this study)				
	Castrocielo, Frosinone	44°29′51″ N 13°41′03″ E	14/08/2017	ITA 2 ^a^	OM912724	H2			
					(this study)				
	Montepulciano,	43°05′23″ N 11°46′47″ E	19/08/2017	ITA 3 ^a^	OM912725	H2			
	Siena				(this study)				
	Cesano, Rome	42°03′50″ N 12°19′44″ E	7/07/2020	ITCE 4 ^a^	MZ398134	H2			
					(Marini et al., 2021)				
				TOHA 2 ^b^	MW892618.1	H2	TOHA 2 cl1, cl3, cl6 ^b^ clones	OM948702-4	H8
					(Kashefi et al., 2021)			(this study)	
							TOHA 2 cl5, 12, 15 ^b^ clones	OM948705-7	H9
								(this study)	
	Bari	41°06′38″ N 16°52′55″ E	31/10/2017				Ailbud ^c^	OM948708	H2
								(this study)	
							Ailleaf ^c^	OM948709	H2
								(this study)	
Croatia	Karlobag	44°31′25″ N 15°04′45″ E	9/08/2017	CRO 1 ^a^	OM912726	H2			
					(this study)				
Greece	Thessaloniki	40°29′49″ N 22°59′19″ E	12/08/2017	GRE 1 ^a^	OM912727	H1	GRE 1 ^a^	OM948710	H6
					(this study)			(this study)	
		40°34′15″ N 22°59′11″ E	20/08/2020	AFS 1 ^b^	OM912728	H1	AFS 1.0 ^b^	OM948711	H5
					(this study)			(this study)	
							AFS 1.1 ^b^	OM948712	H6
								(this study)	
Serbia	New Belgrade	44°49′31″ N 20°25′22″ E	10/05/2017	SER 1 ^a^	OM912729	H1	SER 1 ^a^	OM948713	H6
					(this study)			(this study)	
	Zemun	44°50′33″ N 20°24′19″ E	27/02/2018	SER 2 ^a,^*	OM912730	H1			
					(this study)				
		45°22′53″ N 20°43ʹ37″ E	9/09/2018	SER 3 ^a^	OM912731	H1	SER 3 ^a^	OM948714	H6
					(this study)			(this study)	
Hungary	Palmonostora	46°41′36″ N 19°56′07″ E	2/09/2018	HUN 1 ^a^	OM912732	H1			
					(this study)				
	Lakitelek	46°50′49″ N 19°58′43″ E	6/09/2018	HUN 2 ^a^	OM912733	H1			
					(this study)				
Romania	Timisoara	45°45′09″ N 21°12′38″ E	1/09/2018	ROM 1 ^a^	OM912734	H1	ROM 1 ^a^	OM948715	H6
					(this study)			(this study)	
Bulgaria	Nessebar	42°39′37″ N 27°44′07″ E	12/08/2020	BUL 1 ^b^	OM912735	H1	BUL 1.0 ^b^	OM948716	H6
					(this study)			(this study)	
							BUL 1.1 ^b^	OM948717	H5
								(this study)	
Austria	Vienna	48°13′56″ N 16°25′02″ E	6/07/2017	AUS 1 ^a^	OM912736	H5	AUS 1.0 ^a^	OM948718 (this study)	H3
					(this study)		AUS 1.1 ^a^	OM948719	H4
								(this study)	
Slovenia	Ilirska Bistrica	45°30′54″ N 14°09′29″ E	6/08/2017	SLO 1 ^a^	OM912737	H5	SLO 1.0 ^a^	OM948720	H3
					(this study)			(this study)	
							SLO 1.1 ^a^	OM948721	H4
								(this study)	
France	Colombes	48°55′23″ N 2°13′57″ E	26/05/2020	Colombes3 ^b^	MW892615	H3	Colombes cl7 ^b^ clones	OM948722	H6
					(Kashefi et al., 2021)			(this study)	
							Colombes cl8, cl12, cl14, cl15 ^b^ clones	OM948723-6	H7
								(this study)	
							Colombes cl11 ^b^ clones	OM948727	H8
								(this study)	
				Colombes7 ^b^	MW892616	H1			
					(Kashefi et al., 2021)				
				Colombes8 ^b^	MW892617	H4			
					(Kashefi et al., 2021)				
	Viols le Fort	43°44′45″ N 3°42′31″ E	4/08/2020	TOHA 5 ^b^	MW892619	H1	TOHA 5 ^b^	OM948728	H6
					(Kashefi et al., 2021)			(this study)	
	Manosque	43°48′31″ N 5°48′47″ E	9/09/2020	TOHA 6 ^b^	MW892620	H1	TOHA 6 ^b^	OM948729	H6
					(Kashefi et al., 2021)			(this study)	
	Vinon sur Verdon	43°43′50″ N 5°48′43″ E	10/09/2020	TOHA 7 ^b^	MW892621	H1	TOHA 7 ^b^	OM948730	H6
					(Kashefi et al., 2021)			(this study)	
	Sisteron	44°10′15″ N 5°56′55″ E	10/09/2020	TOHA 8 ^b^	MW892622	H1	TOHA 8 ^b^	OM948731	H6
					(Kashefi et al., 2021)			(this study)	

^a^ DNA archived at IPPE; ^b^ DNA archived at EBCL; ^c^ DNA archived at UBI; * deutogyne females.

**Table 2:** *CO1* pairwise distance matrix with uncorrected *p* distances shown as percentages (above the diagonal) and K2P distances (under the diagonal) between *Aculus mosoniensis*, *Aculops taihangensis,* and two congeners (*Aculus ichnocarpae* and *Aculus amygdali*), and within *Aculus mosoniensis*, and *Aculops taihangensis* (in the diagonal). Data are expressed as mean in % ± S.E.

	*Aculops taihangensis* *n* = 12	*Aculus mosoniensis* *n =* 24	*Aculus ichnocarpae* *n =* 1	*Aculus amygdali* *n =* 1
*Aculops taihangensis* *n =* 12	*p*: 5.62 ± 0.59 K2P: 6.23 ± 0.74	7.68 ± 0.76	22.55 ± 1.52	24.25 ± 1.51
*Aculus mosoniensis* *n =* 24	8.49 ± 0.99	*p*: 2.38 ± 0.28 K2P: 2.59 ± 0.32	23.03 ± 1.59	24.78 ± 1.61
*Aculus ichnocarpae* *n =* 1	27.06 ± 2.22	27.80 ± 2.32		24.62 ± 1.63
*Aculus amygdali* *n =* 1	29.74 ± 2.41	30.57 ± 2.57	30.37 ± 2.68	

**Table 3:** ITS1 pairwise distance matrix with uncorrected *p* distances shown as percentages (above the diagonal) and K2P distances (under the diagonal) within *Aculus mosoniensis*; between *Aculus mosoniensis*, *Aculops taihangensis,* and one congener (*Aculus cercidis*); and within *Aculus mosoniensis* (in the diagonal). Data are expressed as mean ± S.E.

	*Aculops taihangensis* *n =* 1	*Aculus mosoniensis* *n =* 44	*Aculus cercidis* *n =* 1
*Aculops taihangensis* *n =* 1		2.26 ± 0.52	37.02 ± 2.38
*Aculus mosoniensis* *n =* 44	2.31 ± 0.54	*p*: 2.26 ± 0.48 K2P: 2.32 ± 0.42	37.10 ± 2.44
*Aculus cercidis* *n =* 1	51.53 ± 4.7	51.65 ± 4.68	

## Data Availability

Data are contained within the article, whereas the new sequences have been deposited in the Genbank repository.
